# Effect of Arginine and nano-hydroxyapatite application on the hypersensitivity and color change of bleached enamel: A randomized controlled clinical trial

**DOI:** 10.4317/jced.59423

**Published:** 2022-06-01

**Authors:** Lamiaa M. Moharam, Sherif Khadr, Ahmed Abdou, Shaymaa M. Nagi

**Affiliations:** 1 Restorative and Dental Materials Department, National Research Centre, Giza, Egypt; 2 Conservative Dentistry Department, Faculty of Oral and Dental Medicine, Future University, Egypt; 3 Prosthodontic Dentistry Department, Faculty of Dentistry, King Salman International University, Egypt

## Abstract

**Background:**

To evaluate the effect of 2.5% Arginine and nano-hydroxyapatite (nHA) application on the post-bleaching hypersensitivity (HS) and color change in a randomized controlled trial.

**Material and Methods:**

Sixty-four participants were randomly allocated to four groups (n=16) according to the investigated desensitizing agents (UltraEZ gel, experimental 2.5% Arginine, 2.5% nHA pastes and control (no treatment) groups). An in-office chemical bleaching agent (40% hydrogen peroxide) was used for vital bleaching of the anterior teeth. The desensitizing agents were applied to the assigned groups and left for 30 min then rinsed off the teeth. HS was evaluated using Visual Analogue Scale (VAS) from 0-10, immediate (after bleaching), first seven days, then every week for 3-w. Color change was evaluated using VITAPAN classical A1-D4 at baseline (before bleaching), immediate (after bleaching), and after 1,2, 3-w.

**Results:**

HS results showed statistically insignificant difference between the tested groups at day-1 and -2. All groups showed no HS (0) at/and after day-3. There was a statistically significant color change between immediate, 1-w and 2-w and baseline results for investigated materials. No statistically significant difference was recorded between the baseline and 3-w for the investigated materials. Both Arginine and nHA groups showed a higher color change compared to UltraEZ group.

**Conclusions:**

Arginine and nHA present a potential treatment modality for the post-bleaching HS without jeopardizing the bleaching efficiency.

** Key words:**Arginine, nano-hydroxyapatite, bleached enamel, hypersensitivity, color change, clinical trial.

## Introduction

Aesthetic dentistry is at great demand nowadays as many patients are seeking for bleaching of their teeth. Bleaching is a non-invasive and convenient treatment for teeth discoloration, which occurs due to intrinsic and extrinsic factors over time. Bleaching is concerned with chemical degradation of pigments molecules within tooth structure or at its surface. The active ingredient in most bleaching kits is hydrogen peroxide, which can be also delivered in the form of carbamide peroxide (1).

Hydrogen peroxide is an effective oxidizing agent which produces unstable free radicles, that bind to the dark organic pigment molecules breaking them into small, less-intense pigmented fragments, absorbing and reflecting less light giving the “whitening effect” of the bleached teeth (1). Several patients favor in-office bleaching technique due to its quick and satisfying aesthetic outcomes. Hypersensitivity (HS) is the most common adverse effect of vital teeth bleaching, with 15-78% incidence (2). It was suggested that peroxide can pass to the pulp through enamel and dentin creating an inflammatory reaction or directly stimulating neural receptors triggering pain sensation (3). Different approaches with varying rates of success have been advocated to reduce teeth sensitivity, such as decreasing the rate of application, using different desensitizing agents before or after bleaching procedure, desensitizing agent incorporation into the bleaching gel, and decreasing the concentration of bleaching agent. In this context, the efficacy of desensitizing agents to prevent and treat post-bleaching HS has been investigated in many clinical trials offering promising results (4,5). Fluoride is a substantial desensitizing agent, and this might be related to the deposition of CaF crystals within dentin, consequently causing a reduction in the functional diameter of the dentinal tubules (6), which represents a direct passage that allows for hydrogen peroxide diffusion to the pulp. Hypothetically, this deposition could decrease hydrogen peroxide diffusion into the pulp via decreasing dentinal tubules permeability, with no probable effect on the oxidizing power of the active whitening agent (7). Moreover, potassium nitrate was proven to have potent analgesic or anesthetic effect on the nerve endings and nerve fibers. Its mechanism of action depends on prohibiting the repolarization of the excited nerve fibers after their preliminary depolarization in the sequence of the pain signal (8).

Arginine is a natural amino acid that represents one of the constituents of the saliva. The protective effect of the dental hard tissues by the action of Arginine products has been issued for several years. Pro-Argin toothpastes (8% Arginine) and mouthwashes (0.8% Arginine) are effective in treatment of dentin HS (9). On the other hand, nano-sized hydroxyapatite (nHA) has a similar morphology and crystalline structure to the apatite crystal of tooth enamel (10). Therefore, it presents an efficient biomimetic repair for demineralized enamel surface (11). Amin *et al*. (12) indicated that commercially available pastes of nHA can efficiently reduce dentin HS when used as desensitizing agents for 6 months. Consequently, it was concluded that a commercial nHA-based toothpaste showed a complete/partial occlusion of dentinal tubules with frequent use for 6-m, thus reducing the dentin HS (13).

Shade assessment of different esthetic restorations as well as the tooth structure represents a serious challenge in esthetic dentistry. Visual assessment of the shade using commercial shade guides is the most used technique. However, it has been considered a subjective method for shade evaluation. On the other hand, it can be affected by several factors such as surrounding light conditions, color blindness, eye fatigue and the experience of the clinician (14). Vita Classical shade guide was used in the present study to assess color change of the bleached enamel to inspect the possibility of using different desensitizing agents that might improve or undermine the bleaching effectiveness. Thus, this *in vivo* study aimed to compare the effect of experimental desensitizing agents based on 2.5% Arginine and 2.5% nHA on the HS and color stability of bleached enamel. The null hypothesis was that the different tested desensitizing agents would have a similar effect on HS and the color change of the bleached teeth.

## Material and Methods

-Ethical aspects

This clinical trial was registered in www.clinicaltrials.gov database, under protocol NCT04875000. The trial followed the Consolidated Standards of Reporting Trials (CONSORT) Statement (15). All participants signed an informed consent before joining this study, in full accordance with the World Medical Association Declaration of Helsinki (16). The study took place at the outpatient dental clinic of the National Research Centre. Ethical approval was obtained from the Medical Research Ethics Committee (MREC) - National Research Centre (NRC), Egypt (Ref number: 2433042021).

-Sample size calculation

The primary outcome of the study was to evaluate post-bleaching HS for the participants over 3-w follow-up period. Sample size was estimated from the data extracted from *Pi*ntado-Palomino *et al*. (5) A large effect size for the materials investigated (0.7) and (1.2) for the follow-up periods, and their interaction also showed a large effect size (2). The power of the study achieved 96% when a total of 13 patients were assigned for each group. A 15% increase in the patient number in each group (n=16) was determined to accommodate for any possible patients’ drop-out.

Eligibility criteria: Sixty-four participants with ages ranged from (18-55y) were selected to fulfil the following inclusion and exclusion criteria.

Inclusion criteria: Participants with good general, oral health and did not receive any bleaching procedure, caries-free maxillary six anterior teeth without restorations and with no periodontal diseases. Moreover, participants who were willing to assess and sign the informed consent form were included in the study.

Exclusion Criteria: Any participants with non-vital anterior teeth, anterior prosthesis, or orthodontics brackets. Participants suffering from tetracycline stains, fluorosis, or smoking participants. Participants with erosion, dentin exposure, cracked teeth or any other pathologic condition that may elicit teeth sensitivity, or those taking analgesic or anti-inflammatory drugs were excluded from the study.

-Study design, randomization, and allocation 

Sixty-four participants (25 males and 39 females), were enrolled and randomly allocated to four groups (n=16), representing the investigated desensitizing agents (UltraEZ desensitizing gel (Ultradent Products, Inc., South Jordan, UT, USA); 2.5% Arginine; 2.5% nHA pastes and the control (no treatment) groups). In this randomized controlled clinical trial, randomization and allocation of the selected subjects into the respective groups were performed through www.random.org. All participants were blinded yet the operators were not blinded to the application of the desensitizing agents.

-Bleaching procedures

All participants had their maxillary and mandibular anterior teeth polished. Only the maxillary anterior teeth were included in the study, however the mandibular anterior teeth were bleached for patients’ satisfaction. Opalescence™ Boost™ 40% Tooth Whitening System (Ultradent Products, Inc., South Jordan, Utah, USA) in-office chemical bleaching agent was used according to its manufacturer’s instructions. Ultradent IsoBlock™ bite block and self-supporting lip/cheek retractors were placed for soft tissue isolation. OpalDam resin barrier was applied along the gingival margin of the teeth and extended almost 0.5 mm onto enamel surface, forming a continuous layer of 1.5-2 mm thickness. Then it was light cured for 20 s. A 0.5-1 mm thick layer of the bleaching gel was applied to the labial surfaces, left undisturbed for 20 min, then rinsed-off and the teeth were dried. Bleaching procedure was repeated two times for each patient.

-Preparation of Arginine and nHA pastes

Twenty-five g of commercial Arginine (Sigma-Aldrich, St. Louis, MO, USA), and nHA powder (Sigma-Aldrich, St. Louis, MO, USA) were weighed using a digital balance then mixed with 10 ml of distilled water to obtain 2.5% Arginine and nHA pastes.

-Application of the desensitizing agents

Control group received no desensitizing treatment after bleaching procedure. On the other hand, UltraEZ gel (0.11 wt% fluoride ions and 3 % potassium nitrate) was applied to bleached teeth of the assigned test group, using special trays in a generous amount to the labial surfaces and left undisturbed for 30 min then the gel was removed with cotton rolls and the patients were instructed to rinse thoroughly with water. The 2.5% Arginine and nHA pastes were applied to bleached teeth, same way as UltraEZ gel and left undisturbed for 30 min. The patients were instructed to rinse thoroughly with water.

-Hypersensitivity (HS) evaluation

Post-bleaching HS of the bleached teeth was recorded immediately after bleaching (baseline) then following the application of desensitizing agents (immediate) using the Visual Analogue Scale (VAS). Where, the readings were from 0-10. Zero denoted no sensitivity, 1-3 was presented as mild, 4-7 was presented as moderate and 8-10 was presented as severe sensitivity. After that, HS was recorded on daily bases for the first week by the participants who were asked to bring their records in the follow-up sessions. Then the post-bleaching HS was evaluated in-office every week for 3-w using the VAS scoring system. All readings were recorded in a self-structured proforma.

-Color change evaluation

**Figure 1:**
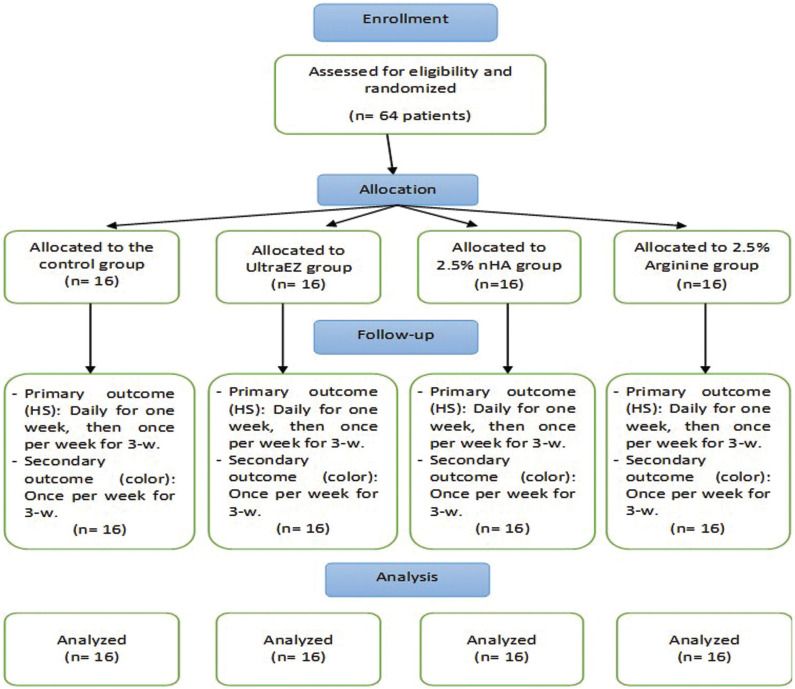
Flowchart of the study according to the Consolidated Standards of Reporting Trial Statement (CONSORT).

Degree of color change was evaluated at baseline (before bleaching), immediate (after bleaching) and for 3-w after the end of the active bleaching. The shades of the middle third of the maxillary incisor teeth were recorded using VITAPAN classical A1-D4® shade guide (VITA Zahnfabrik, H. Rauter GmbH & Co., Bad Säckingen, Germany) under standardized illumination conditions. Sixteen tabs of VITAPAN classical shade guide were arranged from B1 to C4 in a descending value order. Then values were converted into code numbers from 1-16 (17). Color change was calculated by deducting the corresponding number of the determined baseline tab from that of the selected tab at the consequent evaluation according to the arranged shade guide units that occurred toward the lighter end of the value-oriented list of the shade guide. Shade evaluation was done after consensus of three operators with typical color vision verified by Ishiara test according to the detailed conditions proposed by Abdelraouf and Habib (18). Figure 1 shows the CONSORT flowchart of the patients’ enrollment, intervention and data analysis.

-Statistical analysis

Data was explored for normality using Shapiro-Wilk test. It showed non-normal distribution. Data presented as median and interquartile range (Mdn(IQR)). Kruskal Wallis test compared between tested groups for VAS score of HS and color change followed by multiple comparison with Dunn-Bonferroni correction. Friedmann test compared between follow-up periods within each group followed by multiple comparison with Dunn-Bonferroni correction. Linear regression with maximum likelihood estimation presented the effect of different groups while follow-up periods were considered panel data (α=0.05). Statistical analysis was performed using STATA (Version 16; StataCorp LLC, TX, USA).

## Results

**Figure 2:**
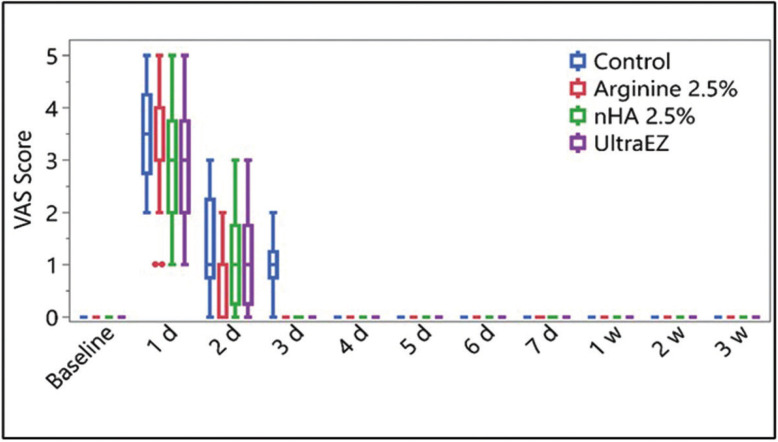
Box and plot showing the VAS score of the hypersensitivity (HS) of the bleached teeth up to 3-w follow-up period for the investigated materials.

A statistically insignificant difference between the tested groups of VAS score of HS at day-1 and day-2 follow-up periods (*p*=0.326 and 0.653 respectively) was presented in Figure 2. Control group showed VAS score of 1(0.75-1.25) at day 3 which was the only group to show pain compared to all other groups (*p* < 0.001). The general effect of using different materials were assessed using the linear regression and showed that both 2.5% Arginine and nHA pastes resulted in a significant increase of the color change of the bleached teeth (ΔSGU) at (*p*< 0.001); 2.5% Arginine paste showed an increase of 1.5 in ΔSGU while, 2.5% nHA paste showed an increase of 1.4 as shown in Table 1. The effect of the investigated materials on the color (SGU) of bleached teeth for 3-w follow up-period was presented in Table 2. There was a statistically significant effect on color between immediate, 1-w and 2-w follow-up periods and the baseline reading the three investigated materials. While no statistically significant difference was recorded between the baseline readings and 3-w for the three investigated materials. No statistically significant difference was recorded between 2.5% Arginine and nHA groups. While there was a statistically significant difference between UltraEZ group and both 2.5% Arginine and nHA groups.

**Table 1:**
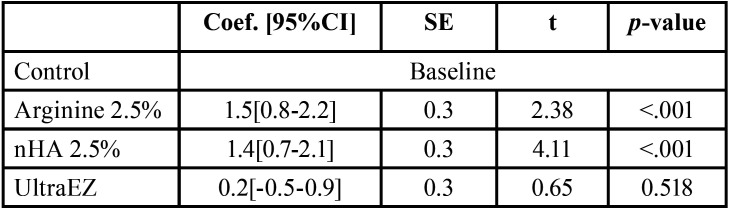
Linear regression results of the effect of the investigated materials on the change in color (ΔSGU) of the bleached teeth.

**Table 2:**
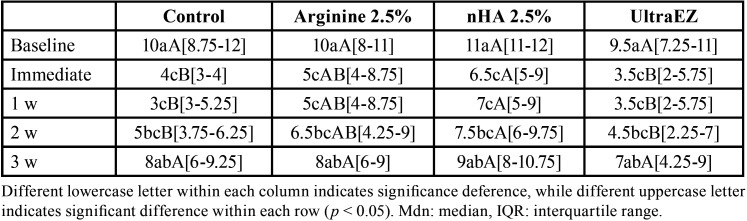
Color (SGU) of the bleached teeth over the 3-w follow-up periods for the investigated materials (Mdn[IQR]).

## Discussion

Application of desensitizing agents following teeth bleaching is a relevant procedure. In this context, the effect of application of experimental desensitizing agents on HS and color change of the bleached enamel was evaluated. The tested null hypothesis was partially accepted because the concurrent application of the three investigated desensitizing agents showed a statistically insignificant effect on the post-bleaching HS. Yet, the investigated desensitizing agents had a statistically significant effect on the color change of the bleached enamel. Regarding previous research, one could deliberate that the bleaching-related tooth sensitivity is a common finding that can be detected among different patients of different backgrounds (4). Moreover, most of the patients were demonstrated to experience varying levels of HS that can be endured during and after the bleaching procedure (19).

The present study showed that UltraEZ gel displayed a statistically insignificant difference of post-bleaching HS from those treated with the experimental 2.5% Arginine and nHA pastes at day-1 and day-2. This could be owed to the comparable desensitizing potential of the three investigated desensitizing agents on the bleached, and hence demineralized enamel surface. Nevertheless, the desensitizing agents are considered as potent obliterating agents that can efficiently prevent the post-bleaching HS (5,12). Da Silva *et al*. (20) concluded that the application of the different desensitizing agents can successfully overcome the ordeal of post-bleaching HS. As these obliterating agents were found to enhance the enamel mineral content, impeding H2O2 infiltration to the nerve endings and thereby HS is much reduced or even eliminated completely. UltraEZ gel is composed of 0.11% by weight fluoride ions and 3% potassium nitrate. The later was found to easily penetrate enamel and dentin, infiltrating the dentinal tubules causing nerve depolarization, thus reducing pain sensation (21). Moreover, application of 5% potassium nitrate in a bleaching tray for 10-30 min was found to decrease the sensitivity in more than 90% of patients included in the clinical study conducted by Haywood *et al*. (22) Kose *et al*. (23) stated that pretreatment with 5% potassium nitrate and 2% NaF prior to bleaching had significantly decreased teeth sensitivity during procedure. Attin *et al*. (24) reported that the application of fluoride enhanced the remineralization of the bleached enamel surface. It was concluded that bleached enamel remineralization was significantly enhanced by NaF application. As demineralized enamel uptakes fluoride more readily than sound enamel. Moreover, deposition of fluoride-containing crystals was evident inside demineralized enamel layer (25). On the other hand, Arginine-containing products are provided with a unique technology that facilitates the diffusion of Arginine through the prisms of the bleached enamel, to substantially obliterate and produce a plug inside patent dentinal tubules, hence permitting a significant relief of post-bleaching HS (26). Such novel technology offers clinically verified profits over long-lasting and quick HS relief. Similarly, it illustrates that Arginine can speed up the tubular occlusion mechanisms via precipitation of a protecting layer upon the dentin surface in contact with bleached enamel (27). Machado *et al*. (28) investigated the desensitizing potential of Arginine on the dental structure. They reported that Arginine has a positively charged guanidinium group which can interact with the negatively charged fluoride ions on the surface of exposed dentin to allow for precipitation of salivary Ca and *P* into the patent dentinal tubules, so that the patent dentinal tubules are blocked, and the flow of the dentinal fluid is decreased. Consequently, it was concluded that a 1.5% Arginine-containing toothpaste with 1450 ppm fluoride in a Ca-base, was more successful in the arrest and remineralization of early enamel lesions in comparison to a 1450 ppm fluoride-containing toothpaste without Arginine (29).

nHA structure is closely like that of teeth, and it can form a protective layer obliterating the openings of dentinal tubules (12). This can explain its positive effect on the post-bleaching HS shown in our results, which could be owed to the ability of nHA to prevent peroxide penetration to the pulp, leading to a significant reduction of HS. Browning *et al*. (4) agreed with our results, they concluded that nHA crystals revealed much better adherence to the demineralized or bleached enamel than the amorphous and large crystals. Additionally, with their smaller size and high area/volume ratio, outstanding bioactivity, and biocompatibility, nHA particles are more likely anticipated to be exceptional materials in HS treatment (4). Narmatha *et al*. (11) demonstrated that application of 1% nHA-containing toothpaste had significantly decreased the overall HS for the participants when related to potassium nitrate. They attributed their finding to dentinal tubules occlusion by the action of the nHA crystals as well as the development of a new acid-resistant biomimetic protective layer.

With respect to color change, the present study used Vita Classical shade guide to evaluate and assess the possible color change of the bleached enamel to inspect the possibility of using the investigated desensitizing agents might improve or undermine the bleaching effectiveness. Colorimeters and spectrophotometers have been mainly used in research and not in the clinical practice (14). This could be related to the relatively high cost as well as the complexity of such equipment and, to the difficulty in using them in *in-vivo* conditions. Up till now, the relation between visual and instrumental shade matching, and the studies’ results, have been indecisive. Inconsistencies of the parameters of color measuring devices or matching teeth to shade systems have been indicated in research. Hence, further investigations are needed to determine the consistency and reliability of using these instruments before they could be merged into routine clinical use (14). Moreover, Parameswaran *et al*. (14) concluded that there were statistically significant differences between the visual and instrumental color matching methods. Nevertheless, the investigated visual method using the shade guides had yielded more accurate results than the instrumental methods using spectrophotometer. Spectrophotometer showed far better interrater agreement scores irrespective of the shade guide used in the study. Yet, the visual shade matching method is subjective, but it was not inferior and should not be underrated in the clinical situations. However, it is recommended to do judicious combination of both techniques to attain a successful and esthetic outcome. The findings of the present study indicated that the darker the baseline tooth color, the higher the degree of bleaching. In this regard, the bleaching results obtained in this study was analogous to those reported in further clinical trial (30). Consequently, desensitizing agents’ application in the current study did not alter the bleaching outcome. Browning *et al*. (4) concluded that nHA desensitizing agent had no significant effect on the bleached enamel color shades after 6-w which was in consistence with our findings.

In terms of color change, one can estimate that alterations in the enamel structure due to the bleaching procedure can be maintained by the application of the desensitizing agents leading to an inconsequential color change of the bleached teeth.

## Conclusions

Regarding the limitations of the present clinical study, it can be concluded that Arginine and nHA present a potential and a promising treatment modality for post-bleaching HS without jeopardizing the bleaching efficiency.
